# Layered-Expanded Mesostructured Silicas: Generalized Synthesis and Functionalization

**DOI:** 10.3390/nano8100817

**Published:** 2018-10-11

**Authors:** Pedro Burguete, José Manuel Morales, Lorenzo Fernández, Jamal El Haskouri, Julio Latorre, Carmen Guillem, Francisco Pérez-Pla, Ana Cros, Daniel Beltrán, Aurelio Beltrán, Pedro Amorós

**Affiliations:** Institut de Ciència dels Materials, Universitat de València (ICMUV), P.O. Box 22085, 46071 Valencia, Spain; pedro.burguete@uv.es (P.B.); J.Manuel.Morales@uv.es (J.M.M.); lorenzo.fernandez@uv.es (L.F.); haskouri@uv.es (J.E.H.); julio.latorre@uv.es (J.L.); carmen.guillem@uv.es (C.G.); francisco.perez@uv.es (F.P.-P.); ana.cros@uv.es (A.C.); daniel.beltran@uv.es (D.B.); Aurelio.beltran@uv.es (A.B.)

**Keywords:** layered materials, amorphous silica, functionalization, dispersion

## Abstract

Mesostructured layered silicas have been prepared through a surfactant-assisted procedure using neutral alkylamines as templates and starting from atrane complexes as hydrolytic inorganic precursors. By adjusting the synthetic parameters, this kinetically controlled reproducible one-pot method allows for obtaining both pure and functionalized (inorganic or organically) lamellar silica frameworks. These are easily deconstructed and built up again, which provides a simple way for expanding the interlamellar space. The materials present high dispersibility, which results in stable colloidal suspensions.

## 1. Introduction

Materials derived from layered inorganic solids continue to attract great interest [1,2,3,4,5,6,7]. There is a diversity of layered solids (silicates, clay minerals, and LDH, among others) that can be tailored to promote specific properties so that they may act as “nanomaterials or as nanoreactors for fabrication of nanospecies, nanoparticles, or nanodevices” [8]. Although many of these solids are abundant in nature, native minerals pose drawbacks (impurities, random and variable composition) that condition their use in a simple way. Nevertheless, the pure materials can usually be obtained by effective syntheses, regardless of possible difficulties (multi-step preparations, including expansion of the interlamellar space) [9]. 

Among the layered solids, those whose skeletons consist solely of [SiO_4_] tetrahedra include different crystalline sodium silicates and some species of silica. While the chemistry and applications of layered silicates (and materials designed from them) have been thoroughly investigated [4,5,6,7], lamellar silicas have attracted comparatively little attention. Actually, the so-called lamellar silicas are, in general, hybrid materials hosting organic species in the interlayer space of mesoscopically-ordered 2D oligosilicic entities. In practice, the organic guest species act as templates directing the assembly of the siliceous network to the layered array [10,11]. The most widely studied among these lamellar silicas is the MCM-50 phase [12,13,14,15,16], which belongs to the family of the M41S materials [17,18]. Typically, the synthesis of the MCM-50 phase is carried out under hydrothermal conditions in basic medium using alkoxysilanes as silica precursors, and an excess of a cationic quaternary alkylammonium species as structural directing agent [11,12,15,18]. On the other hand, it has been described that related lamellar phases can be prepared with the assistance of other surfactants [19,20,21,22,23,24]. Without prejudice to this, the attention paid to the MCM-50 and related lamellar silicas has been much less than that given to other M41S materials (mainly MCM-41 [25]). Moreover, although 2D atomic periodicity has been considered in some cases [13,16], the siliceous frameworks in these hybrids are typically amorphous. This might be disadvantageous when compared with the use of crystalline layered silicates networks with regard to a variety of possible applications [4,5]. Despite this, order is not always an essential requirement [26] and, sometimes, it can be circumvented in exchange for getting procedural advantages, for instance, when the final application requires good dispersibility of plate-like siliceous nanoparticles [27]. Thereby, the usefulness of polymer-clay or layered silicate nanocomposites in a diversity of applications is well known [2]. The goal of this siliceous nanocomposites production “is to uniformly disperse and distribute the inorganic components, initially composed of aggregates of stacks of parallel layers, within the polymer” [1]. In any case, the preparation of the siliceous fillers requires more or less troublesome multi-step processes [4,5,28,29].

After reviewing a diversity of publications concerning the synthesis of lamellar phases, different effects attributed to a variety of factors (sometimes in a contradictory way) have been found. Among others, the nature and concentration of surfactant, surfactant to silicon ratio, silicon source, pH, temperature and time of processing (solvothermal or not), aging, the presence of cosolvents in the reaction medium, even the order of addition of the reagents, or the stirring rate of the reaction mixture can be mentioned. This work reports, with the aim to simplify the problem, a simple reproducible method for the direct growth (one-pot preparation) of new silica-based layered micro/nanocharges combining high purity, organic and/or inorganic functionalization, and easy interlayer expansion (to promote delamination). 

Our group has proven expertise in the surfactant-assisted synthesis of a diversity of mesoporous material compositions [30,31,32,33,34,35], displaying different textural features [36,37,38], using a hydroalcoholic reaction medium allowing the existence of “atranes” (i.e., complexes that include triethanolamine-related ligand species). These act as hydrolytic precursors of the inorganic entities whose assembling with the template agent (micelles) initiates the formation of mesostructured particles [37]. We will benefit here from the versatility of the so-called “atrane route”, which has been detailed elsewhere [37,39]. 

## 2. Materials and Methods 

### 2.1. Synthesis

Regardless of the mechanistic details, the surfactant-assisted mesostructure formation implies the cooperative assembling of organic (micelles) and inorganic (oligomers) supramolecular moieties to yield hybrid composites in which each component comes from the development of its own chemistry [37]. On this basis, we have optimized our preparative procedure, paying attention to those factors of the surfactant chemistry favoring the formation of lamellar mesophases, while maintaining the usual conditions concerning the inorganic counterpart. Thus, the main procedural change with regard to the classic “atrane route” [37,40] is the replacement of cationic trimethylammonium surfactants by neutral primary alkylamines in comparatively high concentrations. We will return to this point below.

### 2.2. Chemicals

All the synthesis reagents were analytically pure, and were used as received from Aldrich (tetraethyl orthosilicate [TEOS], 2,2′,2′′-nitrilotriethanol or triethanolamine [N(CH_2_-CH_2_-OH)_3_, hereinafter TEAH3], octadecylamine (C18), hexadecylamine (C16), tetradecylamine (C14), dodecylamine (C12), decylamine (C10), octylamine (C8), aluminum tri-sec-butoxide and 5,6-epoxyhexyltriethoxyslane, ethanol, DMF, sodium hydroxide, and hydrochloric acid).

### 2.3. Preparative Procedure

All the lamellar pure silicas described herein were synthesized using the “*modified atrane route*”. The molar ratios of the reagents in the starting solutions were adjusted to the following: 2 Si/7 TEAH3/0.75 Cn/90 H_2_O (where n refers to the number of carbon atoms in the tail of the primary alkylamine surfactant). TEAH3, which was in excess with regard to the amount required to form “silatranes” (mainly in the form of Si(TEA)(TEAH_2_)) [39,40], also acted as a cosolvent. In a typical one-pot synthesis, a mixture of TEOS (6 mL, 0.027 mol) and liquid TEAH3 (12.5 mL, 0.094 mol) was heated at ca. 140 °C for 10 min in order to form silatrane complexes in TEAH3 medium. After cooling down to 110–120 °C, a stoichiometric amount (0.75 Cn/2 Si) of the alkylamine was added while stirring. When the resulting solution reached ca. 80 °C, water (22 mL, 1.222 mol) was added slowly whit vigorous stirring. After a few seconds, a white suspension was formed. This mixture was allowed to age at room temperature for 12 h. The resulting mesostructured powder was separated by filtration, washed with abundant water, and air-dried. Gathered in Table 1 are the experimental values of the interlamellar distances resulting for these UVM-Ln solids (where L stands for layered and n for the number of carbon atoms in the surfactant tail). 

The easy inorganic or organic functionalization of silica is a competitive advantage of the “atrane route” [37,40]. Thus, mesostructured layered aluminosilicates (with Al/Si molar ratios ranging from ca. 1/22 to 1/5; the layered morphology is lost for molar Al contents higher than ca. 20%) were synthesized following the above preparative protocol and using hexadecylamine (C16) as a template. Specifically, the molar ratios of the reagents in the starting solutions were adjusted to 2 (Si+Al)/7 TEAH3/0.75 C16/90 H_2_O. Aluminum was added (as aluminum-tri-sec-butoxide) to the TEOS/TEAH3 mixture prior to heating at ca. 140 °C in order to induce co-hydrolysis and co-condensation of the inorganic oligomers. The resulting mesostructured solids were treated as above, and the corresponding experimental interlamellar distances are listed in Table 2 (Al-UVM-L16). As commented below, EPMA microanalysis indicates that the Al content in the mesostructured solids is close to the nominal value in the starting solutions. As a proof of concept, we also synthesized (under the same protocol) an organically functionalized mesostructured layered silica. An epoxy functionality was inserted into the mesostructured from 5,6-epoxyhexyltriethoxysilane, using dodecylamine (C12) as a template agent. The molar ratios in the starting solution were 2 Si/0.16 epoxy/7 TEAH3/0.75 C12/90 H_2_O. The epoxy reagent was added to TEOS at the beginning of the synthesis, which then proceeded as described above.

### 2.4. Reactivity Assays by Chemical Exchange of the Surfactant

To evaluate the feasibility of deconstructing and then reconstructing the silica layered frameworks, we proceeded as follows. We started from the UVM-L12 solid, that is to say, a material containing a relatively short tail surfactant (C12). One gram (approximately 0.007 mol, according to TGA data; see below) of this material was suspended in water (40 mL, 2.221 mol). Then, hydrochloric acid (33 mL, 0.395 mol) was slowly added (3 mL min^−1^) under vigorous stirring. After maintaining the suspension under vigorous stirring for 1 h in this highly acidic medium at room temperature, the mesostructure collapsed (XRD) by extraction of the surfactant as a result of an inadequate charge matching. The resulting amorphous (XRD) solid was filtered, washed with water, and air-dried. Once the “collapsed variety” of the UVM-L12 material (silica) was prepared, the layered net was reconstructed by mixing this solid (0.5 g, approximately 0.008 mol) with octadecylamine (C18) (1.1 g, 0.004 mol) in a solution containing water (15 mL, 0.833 mol), ethanol (15 mL, 0.257 mol), and hydrochloric acid (2 mL, 0.024 mol). The mixture was refluxed (at 65 °C) for 48 h. The resulting solid was filtered, washed with water, and dried at room temperature. As commented below, the XRD data show that the interlayer expansion (i.e., the reconstruction of a laminar framework) was effective.

### 2.5. Physical Measurements

All solids were characterized by X-ray powder diffraction (XRD) at low angles (Seifert 3000TT θ-θ; Seifert Systems GmbH, Aldersbach, Germany) using Cu Kα radiation. Patterns were collected in steps of 0.03° (2θ) over the angular range 0.73–10° (2θ) for 10 s per step. TGA/DTA curves were simultaneously recorded with a Setaram Setsys 16/18 thermobalance (Setaram Instrumentation, Caluire, France) under an O_2_ atmosphere flowing at 25 mL per min (heating rate of 5 °C/min). FTIR spectra were collected on a Nicolet 4700 spectrometer (Thermo Fisher Scientific, Waltham, MA USA). The spectra were registered (using self-disks of 1% sample in KBr) at room temperature and the spectrometer was continuously purged with dry air. Transmission electron microscopy (TEM) was carried out with a JEOL-JEM-1010 microscope (JEOL Ltd., Tokyo, Japan) operated at 100 kV (equipped with digital camera MegaView III and “ANALYSIS” software). Some samples were embedded in LR-white resin. Later, ultra-thin cuts (thickness of 60 nm) were made in a Leica Ultracut UC6 equipment (Leica, Wetzlar, Germany) with diamond blade DISTOME. Atomic force microscopy (AFM) measurements were performed using a Nanotec (Nanotec Electronic GmbH & Co. KG, Feldkirchen, Germany) multimode instrument operating in tapping mode. Slow scanning speeds were used to prevent the appearance of artifacts in AFM images. ^29^Si and ^27^Al MAS NMR spectra were recorded on a Varian Unity 300 spectrometer (Varian, Inc., Palo Alto CA, USA). The MAS probe was tuned at 79.5 (^29^Si) and 78.16 (^27^Al) MHz, with a magic angle spinning speed of at least 4.0 KHz. Aluminum functionalized solids were characterized by electron probe microanalysis (EPMA) to determine the Si/Al molar ratio using a Philips SEM-515 instrument (Philips, Amsterdam, Holland). Particle size distribution was determined by using a Malvern Mastersizer 2000 instrument (Malvern Panalytical, Malvern, UK) equipped with a small-volume sample dispersion unit. Ethanol and DMF were used as dispersion media. Each sample was measured in triplicate, accumulating light scattering data for 10 s (in each measurement, as well as in the background). To favor particle dispersion, samples were sonicated for 2 min at 15 W in successive treatments until stabilization of the particle size distribution. 

## 3. Results and Discussion

### 3.1. Synthesis Strategy

In previous publications, both the optimization of experimental conditions and probable mechanisms for the surfactant-assisted synthesis of hexagonal mesostructured/mesoporous silicas by means of the “atrane route” were discussed in detail. By using alkylammonium salts as (cationic) templates, this one-pot procedure allowed for obtaining hexagonal mesophases (under rather soft temperature conditions and short reaction times) at apparent pHs values ranging approximately from 9 (nanoparticulated UVM-7 materials) to 10.5 (microparticulated MCM-41-like phases) [41]. The molar ratio of the reagents in the starting solutions was typically optimized according to 2 Si/7 TEAH3/0.52 CnTMABr/180 H_2_O (where 10 ≤ n ≤ 18), and, when necessary (MCM-41), the pH was adjusted by addition of a solution of NaOH. As mentioned, this work pays attention to those aspects of the surfactant chemistry favoring the formation of lamellar mesophases. Without prejudice to any other factor, there seems to be agreement on the structural relevance of the effective surfactant packing parameter, g = V/a_0_ l. The increase of *g* is related to a reduction of the surface curvature of the surfactant array, which favors lamellar mesophasic arrangements [12,42]. Accordingly, keeping everything else unchanged, the use of neutral surfactants with relatively small head groups (instead of relatively bulky cationic alkylammonium salts) should favor the lamellar packing in the mesostructure by increasing the *g* parameter, as a consequence of the reduction of the head group effective area at the micelle surface, a_0_ [12,43,44]. Therefore, the first synthetic choice was to replace CnTMABr with neutral primary alkylamine surfactants having similar tails, while using “silatranes” as hydrolytic silica precursors. Anyway, it is worth noting that the “neutral templating route” was explored for the first time by Tanev and Pinnavaia, although they obtained hexagonal mesoporous silicas (HMS, related to MCM-41) using TEOS as silica precursor in an ethanol rich medium to solubilize the amines [45,46]. Regardless of mechanistic details, other factors frequently identified as very influential in the structural control by the surfactant of the growing of different silica mesophases are the surfactant concentration, the surfactant/silicon molar ratio in the reaction mixture, and the pH. Thus, high pH values and increasing relative surfactant concentrations should favor the formation of the lamellar mesophase [18,22,41,43].

In order to optimize the “modified atrane route” procedural variables, a series of experiments were carried out. In these experiments, for each one of the primary alkylamines (8 ≤ n ≤ 18), (a) the 2 Si/7 TEAH3 ratio used in the “atrane route” was maintained; and (b) the surfactant to silicon molar ratio (0.125 ≤ x = [Cn/Si] ≤ 1), the water amount (30 ≤ [H_2_O/Si] ≤ 60, in moles), and the pH of the starting solution were successively modified.

**Figure 1:**
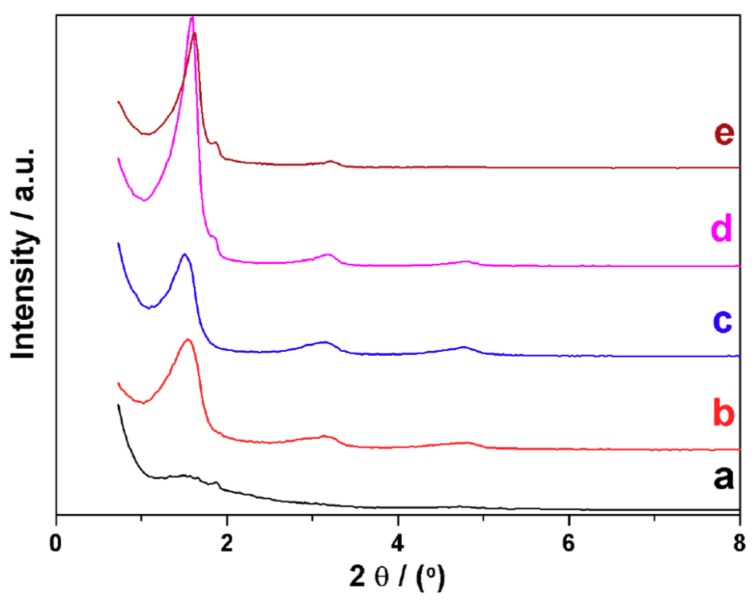
Low-angle X-ray powder diffraction (XRD) patterns of UVM-L16 solids synthesized by using different C16/Si molar ratio: (**a**) 0.125, (**b**) 0.25, (**c**) 0.5, (**d**) 0.75, and (**e**) 1.

Low angle XRD patterns shown in Figure 1 correspond to the as-synthesized solids using hexadecylamine as a template (0.125 ≤ x = [C16/Si] ≤ 1) at the basic pH (ca. 10.5) generated by the addition of the alkylamine [47], and a surfactant concentration resulting from the addition of 45 moles of H_2_O per mol of Si. With the exception of x = 0.125 (ill defined), the patterns are typical of layered compounds. They exhibit a high intensity peak with a *d* spacing between the silica shells (corresponding to the (001) reflection) of approximately 5.5 nm, and at least two higher angle (2θ) peaks consistent with the respective (00l) harmonics. The absence of any diffraction peak in the high-angle domain confirms the amorphous nature of the silica sheets. The intensity decrease, together with the presence of a shoulder on the intense (001) peak for x = 1, is likely related to the adsorption of a certain excess of surfactant [48]. The best pattern resolution (FWHM values), which has been taken as a quality criterion, is associated with the x = 0.75 sample. In practice, the situation changes very little when the amount of water present during hydrolysis is varied in the molar range 30 ≤ [H_2_O/Si] ≤ 60. However, XRD patterns lose definition for dilutions higher or lower than that in the previous experiment ([H_2_O/Si] = 45). On the other hand, in order to evaluate the influence of pH in the evolution of the starting solution, small amounts of concentrated solutions of HCl or NaOH were carefully added after water addition. It is worth noting that the apparent pH of the reaction mixture during the synthesis practically does not alter the resulting *d* spacing value when working in the, approximately, 8.5 ≤ pH ≤ 11.5 range. At pH values lower than 8.5, the C16 micelles and the oligomeric silicic entities do not adequately match to give lamellar mesostructures. On the other hand, the quality of the XRD patterns decreases at pH values higher than that originated by the primary C16 amine (ca. 10.5), which indicates a progressive loss of the mesoscopic ordering in the resulting mesostructures. In short, the above-mentioned synthesis parameters (2 Si/7 TEAH3/0.75 C16/90 H_2_O) were adopted as more adequate for obtaining the lamellar silica mesophase through the “modified atrane route”. As can be noted, this implies using comparatively high concentrations of the neutral primary alkylamine surfactant with regard to the classic “atrane route”. Working at the pH value resulting from the amine itself (ca. 10.5) leads to the formation of lamellar silica mesophases instead of the hexagonal MCM-41 silicas, which were obtained by NaOH addition when using alkylammonium cationic surfactants as templates. It is remarkable that completely analogous results are obtained for all the primary alkylamines (8 ≤ n ≤ 18) used as surfactants (Figure S1), with the only evident difference of the corresponding *d* spacing values (see Table 1).

### 3.2. Characterization

**Figure 2:**
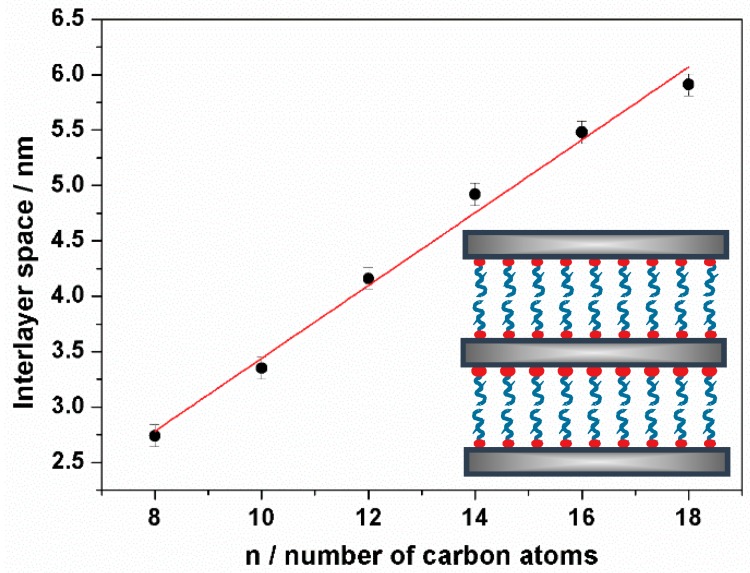
Evolution of the interlayer space vs. the number of carbon atoms in the alkylamine surfactant tail.

The evolution of the *d* spacing value with the number of carbon atoms in the surfactant tail (n) is shown in Figure 2. There is a rather good linear correlation of *d* with n, with a slope of ca. 1.5 nm per each additional carbon atom in the alkylamine chain. This result supports the fact that the surfactant tail length controls the interlamellar distance (Figure S1). This value is slightly larger than that usually associated with all-trans arrays in alkylamines (1.26 nm). In any case, it suggests that the surfactant moieties between the silica layers should be organized as non-inter-digitalized bilayers (without tilting), a disposition that is usually referred to as paraffinic (inset in Figure 2) [49]. The somewhat elevated experimental value might be attributed to factors such as the adsorption of variable water amounts in the silica-surfactant hydrophilic interphase or even to a certain heterogeneity in the surfactant organization.

In line with the above mentioned, the thermal evolution (TGA–DTA, Figures S2 and S3; Table 1) of the different materials shows, in all cases, a relatively small initial weight loss below 100 °C, which might be mainly associated with the elimination of water molecules located among the surfactant heads and the silica layers. On the other hand, surfactant removal always takes place in two different stages partially overlapping. Thus, a first significant weight loss (associated to a sharp endothermic effect) occurs in the ca. 100–200 °C temperature range, which is followed by a second smaller weight loss (endothermic signal) occurring mainly between ca. 200 °C and 600 °C. The experimental weight losses corresponding to the different materials are gathered in Table 1. Without prejudice from those indicated above, the temperature ranges at which the alkylamines are eliminated seem to shift slightly as the tail length of the surfactant increases (there is a certain “delay” in the thermal evolution), which mainly affects the ca. 100–200 °C stage. Indeed, this stage is practically completed below 200 °C for C8, C10, and C12, while this limit temperature progressively increases for C14, C16, and C18. It seems reasonable to relate this “delay” in the thermal evolution with the need for a greater energy contribution to disturb the increasing van der Waals interactions among the alkyl chains as their length increases. Nevertheless, the most significant fact is that, in all cases, the elimination of the surfactant occurs in two stages at sufficiently differentiated temperatures. This result leads us to think of different ways of interaction between the organic portions and the silica layers, with stronger links between the silica surface and those alkylamine chains lost at a higher temperature. The experimental weight losses (Table 1) indicate that the molar ratio between the surfactant amounts eliminated at relatively low and high temperature fits rather well with ca. 1.5 ± 0.2 for materials (exception made of the solid containing the shorter alkylamine (C8), for which this ratio is close to 2). On the other hand, by comparing the total weight losses associated with the surfactant removal for the different materials, we have verified that there is a good correlation between these ratios and those of the molecular masses of the surfactants, which would suggest similar packing densities of the surfactants in the interlayer spaces.

Infrared spectroscopy techniques were previously applied to get information about the interlayer structure in alkylammonium layered silicates [49,50]. It was noted that the strongest aliphatic absorption bands observed in all cases are the methylene modes appearing around 2920, 2852, and 1468 cm^−1^, which can be ascribed to the υ_as_(CH_2_), υ_s_(CH_2_), and δ(CH_2_) vibration modes, respectively. The frequency of the υ_as_(CH_2_) band in these solids shifts from ca. 2930 to 2920 cm^−1^ as the tail length of the surfactant increases. This evolution was related to the order and organization of the surfactant in the interlayer space; from low ordered organic lateral layers or bilayers to ordered paraffin-type bilayers.

**Figure 3:**
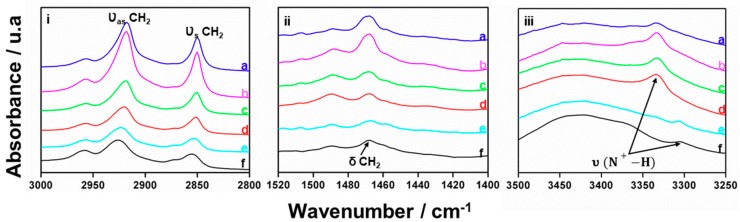
FTIR spectra of samples (**a**) UVM-L18, (**b**) UVM-L16, (**c**) UVM-L14, (**d**) UVM-L12, (**e**) UVM-L10, and (**f**) UVM-L8. (**i**) Stretching C–H modes. (**ii**) Bending H–C–H modes. (**iii**) Stretching N–H bonds.

In the case of our UVM-Ln materials, the location of the υ_as_(CH_2_) band in the corresponding FTIR spectra (Figure 3i) does not seem to be significantly altered by the length of the aliphatic chain of the surfactant. In practice, this band is observed at 2921 ± 3 cm^−1^, without any appraisable regular trend with n. Such a value, closer to the typical of hydrocarbon chains in all-trans conformations (2918 cm^−1^) than to that characteristic (2929 cm^−1^) for highly disordered arrays (liquid-like) [49,51], suggests a relatively high degree of order and a predominance of paraffinic all-trans arrangements of the surfactants in our materials. This organization is also consistent with the observation of the band due to the δ(CH_2_) vibration mode (Figure 3ii). In the spectra of the UVM-Ln solids, this band is centered at 1468 cm^−1^ (exception made of the solid containing C18, for which this band appears at a very slightly higher frequency). In accordance with the argumentation of Vaia et al. [49], “the observed absorption around 1468 cm^−1^ is characteristic of a partially ordered phase where the chains are mobile while maintaining some orientational order”. The FTIR-suggested predominance of paraffinic all-trans arrays of surfactant molecules between the silica layers is in good agreement with their non-inter-digitalized bilayer organization suggested by the XRD results. 

On the other hand, although the aliphatic absorption bands can inform us about the disposition of the surfactant molecules between the silica layers, they cannot provide information concerning the above-mentioned feasibility of different ways of interacting between the organic portions and the silica layers. To gain insight on this last aspect, we have focused our attention in the 3000–3500 cm^−1^ energy range of the FTIR spectra (Figure 3iii). Without prejudice from the expected poor definition of the intense and broad signal that occupies this spectral region (it must include all the N–H and O–H stretching vibrations), we observe, in all cases, a well-defined peak at frequencies in the 3300–3350 cm^−1^ range. In fact, this IR signal (typically assigned to N–H stretching vibrations) can be considered as a fingerprint of protonated amine groups (υ(N^+^-H)) [50,52]. Thus, it is reasonable to conclude that, in all UVM-Ln solids, a part of the alkylamine molecules is cationic. Actually, working at the basic pH values due to the proper amines (ca. 10.5), the oligomeric silicic entities must carry some negative charge on their surface, and an adequate charge matching with the organic counterparts will require partial protonation of the alkylamine portions. Therefore, everything indicates that the mesostructure cohesion is achieved by the effect of both weak hydrogen bonds (between amine molecules and surface silanol groups) and ionic interactions (involving alkylammonium cations [CH_3_(CH_2_)_n_NH_3_^+^] and the anionic silica surface (deprotonated silanol groups, SiO^−^)). If so, we can say that a combination of S^+^I^−^ and S^0^I^0^ mechanisms cooperatively works in the surfactant-assisted mesostructure formation. In fact, these hydrogen-bond versus ionic alkylamine-silica surface interactions would account for the two stages process (surfactant removal) observed in the TGA–DTA experiments: the first weight loss (ca. 100–200 °C) could be associated with the evolution of neutral alkylamine molecules, while that observed at higher temperatures (ca. 200–600 °C) should be the result of the degradation of alkylammonium entities. The nearly constant ratio between neutral and protonated alkylamine molecules (1.5 ± 0.2, except for C8) determined by TGA may be thought of as a consequence of the similar (basic) pH conditions provided by the alkylamines in the hydroalcoholic reaction media. Similar pH conditions must lead to similar densities of SiO^−^ surface groups able to interact with the protonated amine heads of the surfactants. It can be said that this model is in good agreement with recent molecular dynamics simulations on related derivatives, which support both the existence of alkylamine/alkylammonium portions as well as their relative proportion (ca. 60/40 at pH close to 10.5 in our case) [51].

**Figure 4:**
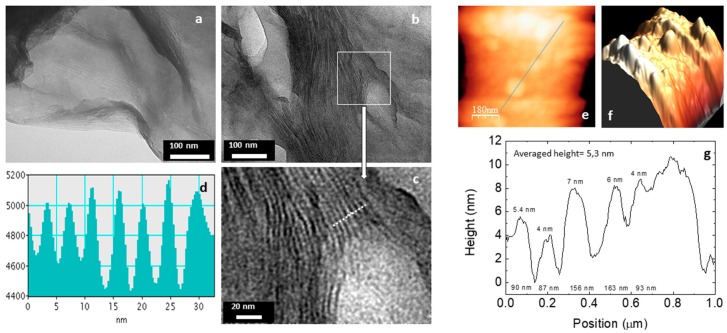
Transmission electron microscopy (TEM) images of (**a**) sample UVM-L18, (**b**) and (**c**) UVM-L18 sample embedded in LR-white resin. (**d**) Line profile of the image intensity of the Si atomic row. Two-dimensional (**e**) and 3D (**f**) Atomic force microscopy (AFM) images of sample UVM-L18. (**g**) Height vs. position along the line indicated in the (**e**) image.

SEM images of UVM-Ln materials show the existence of relatively large micrometer particles/grains in which a certain 2D organization seems to be present (Figure S4), but they are not conclusive. However, TEM images unambiguously confirm the layered morphology of the UVM-Ln solids. In any case, the usual preparation of the samples by simple deposition on an electronic microscopy grid does not provide enough information as a result of preferential orientation of the sheets (perpendicular to the electron beam). A representative micrograph corresponding to the UVM-L18 solid is shown in Figure 4. We can appreciate that the materials are built up from the stacking of micrometric 2D silica particles. We can observe characteristic marks of the layers at the grain edges, but it is not possible to obtain defined information regarding the stacking sequences; the observed marks only allow us to confirm the growth of the particles in terraces. In order to avoid this problem, we prepared composite samples by dispersing the siliceous particles in a polymeric (LR-white resin) matrix. Then, we made cuts using an ultramicrotome. The resulting samples were deposited as usual on a copper grid. TEM images obtained in this way are shown in Figure 4b,c. We observed a good dispersion of the siliceous particles (UVM-L18) in the matrix, which appear as ordered domains involving approximately 10–12 silica sheets. It must be emphasized that such a dispersion was achieved without any optimization with respect to the concentration of the charge or the nature of the polymer employed. Moreover, the line profile of the image intensity of the Si atomic row in the same region (Figure 4d) demonstrates the homogeneity of the interlayer distances, with a repetitive parameter of ca. 6 nm. As can be noted, this value fits very well with that previously calculated by XRD, namely 5.8 nm. A similar value (ca. 5.5 nm) was also estimated by preliminary AFM measurements (Figure 4e,f,g).

**Figure 5:**
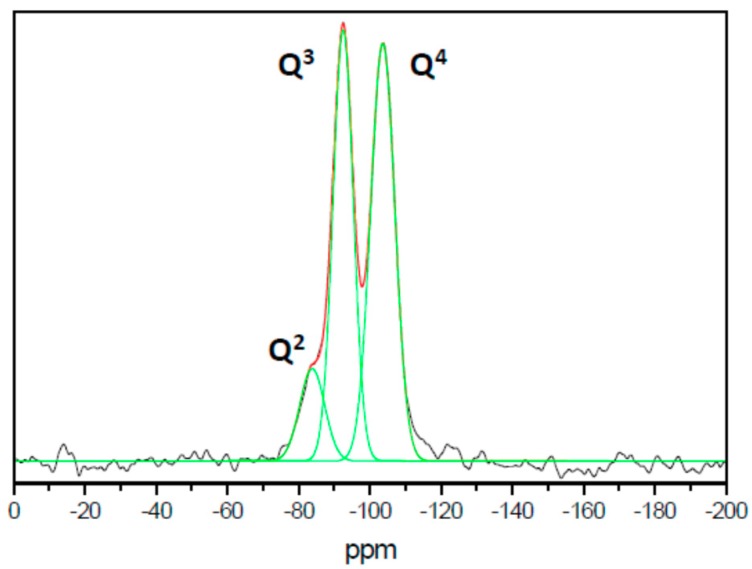
Gaussian deconvolution of the ^29^Si MAS NMR spectrum of sample UVM-L12.

Additional information about our materials can be obtained by means of ^29^Si MAS NMR spectroscopy. A representative spectrum of the UVM-Ln family (corresponding here to UVM-L12) is shown in Figure 5. Gaussian deconvolution of the spectrum displays three peaks at chemical shift values of −83.8, −92.5, and −103.6 ppm, which can be associated with Si environments of the types Q^2^ (relative intensity 11%), Q^3^ (41%), and Q^4^ (48%), respectively (where Q^n^ stands for Si(OH)_4_–n(OSi)_n_, n = 0–4) [53]. By comparison with the spectra of other mesoporous materials of the M41S family [17], it can be highlighted that the signals corresponding to the Q^3^ and Q^4^ sites are sharp, of similar intensities, and, comparatively, they appear rather well resolved. These features have been previously described for other lamellar silica mesophases [13,14,43] and were attributed to the existence of a certain molecular order, that is to say, to the presence of well-defined Si sites. In fact, the resolution of these signals is complete for crystalline layered silicates [54].

### 3.3. Functionalized Materials

Although, a priori, the inorganic/organic functionalization of amorphous silica materials might seem straightforward, there is abundant information in the bibliography relative to the preparative difficulties associated with such a goal [55]. These difficulties (many times resulting in lack of reproducibility) span from the effective incorporation (and amount) of the functional species in the silica framework to its localization and dispersion (homogeneity and/or phase segregation) throughout the material. 

Definitely, when dealing with chemically complex systems (multicomponent), we can better appreciate the efficiency and versatility of the “atrane route”. Hence, to explore the capability of our modified procedure, we prepared a series of Al-containing (typical dopant-element in siliceous materials because of its interest in catalysis) UVM-L16 materials by the simple recipe described in the experimental section. Table 2 summarizes the main physical parameters corresponding to the resulting Al-UVM-L16 materials.

EPMA analyses of the title compounds show a high chemical homogeneity at micrometric level (spot area ca. 1 μm), with a good dispersion of Al and Si atoms. Thus, the solids can be considered as single-phase products and segregation of aluminum oxides can be discarded even for the samples with relatively high Al contents (which is consistent with the absence of peaks in the high-angle region of the corresponding XRD patterns). On the other hand, the Si/Al molar ratio values in the final materials are close to those present in the starting solutions, which suggests that there is no preferential incorporation of aluminum or silicon into the final net.

**Figure 6:**
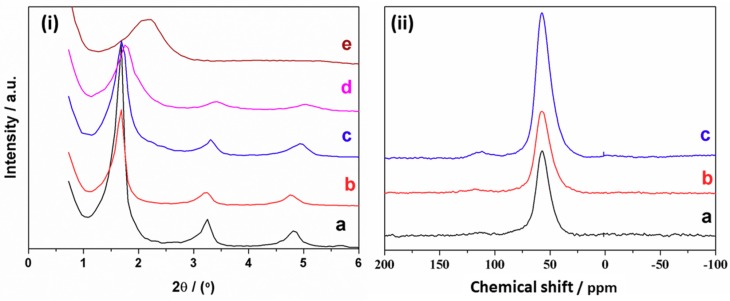
(**i**) Low-angle XRD patterns of samples (**a**) UVM-L16 and the Al modified solids Al-UVM-L16 with different Al content (Si/Al real molar ratio): (**b**) 22, (**c**) 7, (**d**) 5, and (**e**) 3. (**ii**) ^27^Al MAS NMR spectra of Al-UVM-L16 samples with different Al content (Si/Al real molar ratio): (**a**) 22, (**b**) 7, and (**c**) 5.

Low-angle XRD patterns of the Al-UVM-L16 samples are shown in Figure 6i. All solids whose Si/Al molar ratio range from ca. 5 (ca. 17% of Al) to 22 (ca. 4.5% of Al) show diffraction patterns with three (00l) reflections at low 2θ values, which is typical of layered materials well-ordered along the *z* direction. In this compositional range, the inter-lamellar spacing in the materials is constant and very close with that estimated for the pure UVM-L16 solid (ca. 5.48 nm). In contrast, the pattern of the sample richest in Al (Si/Al molar ratio ca. 3; ca. 25% of Al) displays only one broad signal. Therefore, the results indicate that, regardless of the coordination, Al incorporation in the layers does not imply significant changes (or decreasing order) in the silica-based framework up to reach high Al contents (ca. 17% of Al). 

Once the UVM-Ln architecture in the Al-containing materials is defined, it seems relevant to elucidate how the inclusion of Al in the silica matrix occurs. ^27^Al MAS NMR spectra of the Al-UVM-L16 solids (ca. 22 ≥ [Si/Al] ≥ 5 molar ratio compositional range) are shown in Figure 6ii. NMR results confirm the incorporation of Al to Si-sites in the net. Thus, the ^27^Al MAS NMR spectra show essentially only one resonance signal at a chemical shift (δ) of ca. 55 ppm, which is characteristic of tetrahedral Al sites [53]. Moreover, the absence of any signal at ca. 0 ppm allows one to completely discard the presence of octahedral Al environments [53]. The fact that Al incorporation from the starting solution seems to be virtually complete, as well as the fact that this incorporation takes place exclusively in framework positions, can be understood on the basis of the harmonization of the Si and Al reactivity achieved because of the (simultaneous) initial formation of the respective atrane derivatives [40]. In the same way that the modified atrane route allows us to obtain layered Al-UVM-Ln derivatives, it does not seem unrealistic that, as it happens for MCM-41 or UVM-7, a diversity of heteroelements may be incorporated in lamellar UVM-Ln matrices.

Inspired by the way in which the classic “atrane route” was extended for obtaining bimodal porous organosilicas related to the UVM-7 materials [56], we have preliminary explored the possibility of applying the current modified procedure for obtaining organically modified layered silicas. Thus, as a proof of concept, we have synthesized a layered organosilica containing terminal epoxide groups, which might be of interest to covalently connecting to polymers. Our starting solution (see experimental section) contained both TEOS and 5,6-epoxyhexyltriethoxysilane (in the presence of an excess of TEAH3 to generate silatranes), and we selected dodecylamine (C12) as a surfactant. The preparative protocol was the same as that optimized for the UVM-Ln (n = 12) pure silica. The low angle XRD pattern (Figure S5) displays the three first (00l) reflections. While confirming the maintenance of the lamellar arrangement, the experimental interlayer *d* spacing value (4.1 nm) is identical to that of the UVM-L12 pure silica. Therefore, it seems reasonable to conclude that the organic functionalization does not affect the surfactant, which preserves its paraffin-like organization.

**Figure 7:**
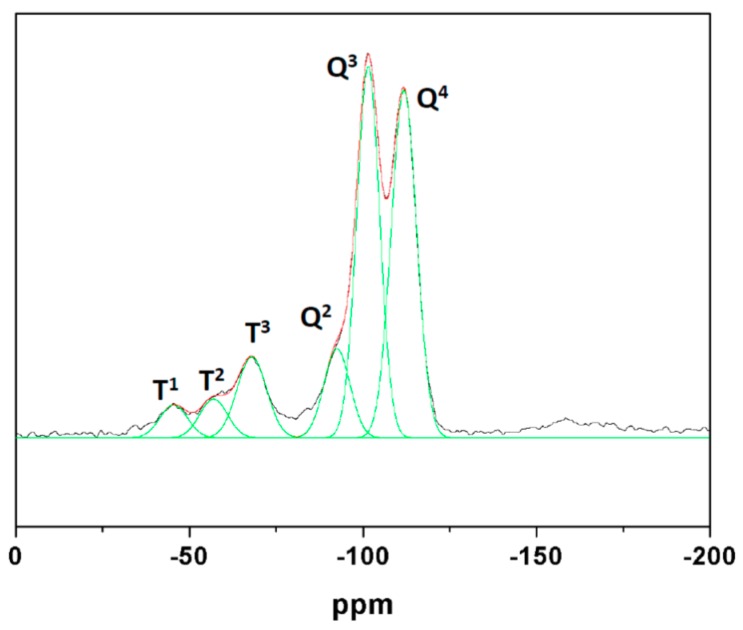
Gaussian deconvolution of the ^29^Si MAS NMR spectrum of Epoxy-UVM-L12 sample.

The ^29^Si MAS NMR spectrum in Figure 7 confirms the incorporation of the organic silane derivatives to the UVM-L12 silica. Thus, deconvolution of the spectrum allows identifying both Q (Q^n^ stands for Si(OH)_4−n_(OSi)_n_, n = 0–4) and T (T^m^ for RSi(OSi)_m_(OH)_3−m_, m = 0–3) Si sites. We can identify up to six Gaussian signals. The Q^n^ sites are located at −92.5 (Q^2^, ca. 10%), −101.5 (Q^3^, ca. 35%), and −111.9 (Q^4^, ca. 37%) ppm, respectively. The T^m^ sites originate signals at −47 (T^1^, ca. 4%), −57 (T^2^, ca. 4%), and −68 (T^3^, ca. 10%) ppm, respectively. As noted, the approximate 1:1 ratio between the Q^3^ and Q^4^ centers remains. However, there is now a somewhat greater overlap between these signals with respect to the case of the pure lamellar silicas, an evidence that can indicate that the incorporation of the epoxy functionality induces a certain loss of molecular order in the silica net. On the other hand, NMR data confirm the efficiency of silane incorporation to the silica. Indeed, whereas the Si(Q)/Si(T) (i.e., TEOS/5,6-epoxyhexyltriethoxysilane) nominal molar ratio in the starting solution is 12.5, the real molar ratio in the functionalized silica is 4.5. This means that a significant enrichment in the organic silane portion has taken place (probably associated with the higher solubility of the pure inorganic silica species). In short, all of this evidence supports the feasibility of the modified atrane route for obtaining layered organosilicas (with a relatively high functionalization degree).

### 3.4. From Layered Mesostructures to Colloids

As suggested above, any potential application of the layered silicas requires easiness to expand the interlamellar space, either to carry out intercalation processes or either to achieve a good delamination and dispersion of the initial aggregates of stacks [57]. Then, we conducted preliminary experiments to verify that the UVM-Ln matrices are suitable materials for this purpose.

**Figure 8:**
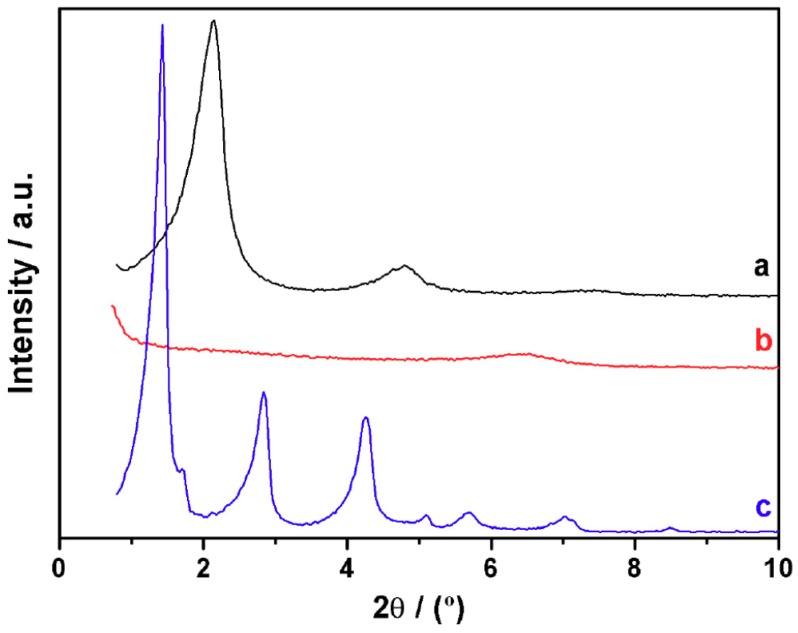
XRD patterns of (**a**) UVM-L12, (**b**) collapsed surfactant-free UVM-L12, and (**c**) rebuild expanded phase using C18 as a surfactant.

In the experimental section, we describe a simple experiment concerning the possibility of delaminate a layered matrix (UVM-L12) and rebuild it in the form of a new layered-expanded mesostructured silica (UVM-L18). Indeed, the C12 surfactant can be removed from the UVM-L12 framework by treating the material in aqueous solution in acid medium. In practice, this treatment results in the disappearance of the intense XRD peaks characteristic of the layered mesophase. The resulting material only creates a low intensity wide signal centered at 1.4 nm (Figure 8). However, by refluxing this solid in a hydro-alcoholic acid medium in the presence of C18, layer-expansion by surfactant intercalation occurs. In fact, three intense (00l) signals are recovered in the XRD pattern of the final solid, and an interlayer space of ca. 5.7 nm can be measured (a value very close to that determined for the solid UVM-L18, 5.8 nm) (Figure 8).

**Figure 9:**
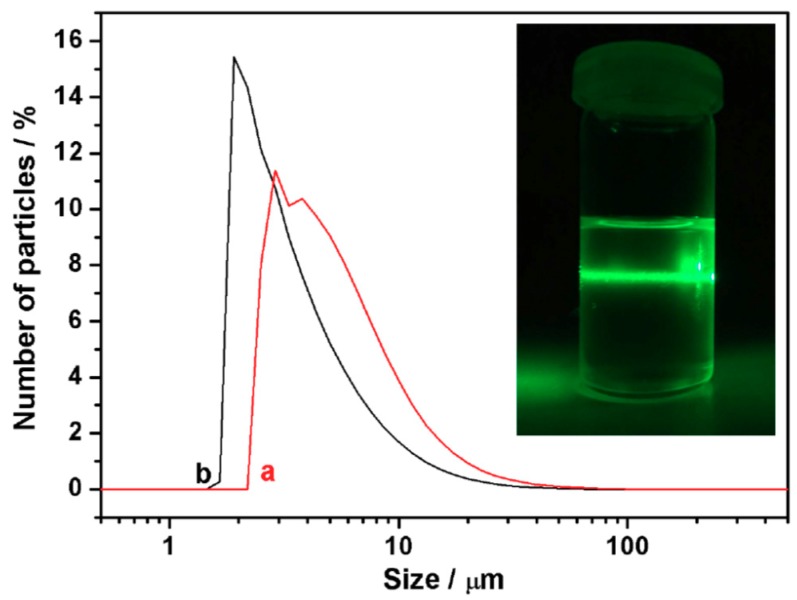
Laser diffraction particle size distribution of UVM-L16 in (**a**) ethanol and (**b**) DMF.

On the other hand, although it exceeds the objectives of this work, a possibility to be explored is the suitability of the UVM-Ln matrices as charges to get useful siliceous–polymer composites. In practice, we have made a first rudimentary (not optimized) approach to this subject by preparing the composite samples (UVM-L18/LR-white resin) that allowed us to observe the UVM-L18 layered morphology by TEM (Figure 4). Without prejudice from that stated above, this result suggests that the (adequately processed) UVM-Ln matrices might be incorporated as charges to polymers. In any case, achieving a uniform distribution of the charges within polymers will require, first of all, a good dispersibility of the siliceous matrix. We conducted preliminary experiments to initially evaluate this capability. Thus, the UVM-Ln derivatives could be incorporated into composites by the solution blending method, which has been widely used (in some cases, more effectively than the melt blending protocol) [58]. With this aim, it is necessary to select solvents favoring the dispersion of the silica filler and the subsequent polymerization process. In our preliminary dispersibility experiments, we used ethanol and DMF (two typical solvents) and the silica matrix assayed has been UVM-L16. After sonication, suspensions containing 0.01 g of UVM-L16 in 50 mL of the solvents have been analyzed by laser diffraction. As shown in Figure 9, in both solvents, the resulting particle size distribution ranges from ca. 2 to 80 μm. Nevertheless, the dominant particle size is always below ca. 10–20 μm, while the maxima in the distribution curves appear at 2.5 (DMF) and 3.2 (ethanol) μm. These last values would correspond to the majority sizes of the resulting deconstructed layers (or their aggregates). The suspensions remain stable over 3–4 h, at least, and light scattering by the colloidal particles (Tyndall effect) is clearly observable (Figure 9). TEM images obtained by suspension gouts deposition on Cu grids (Figure S6) do not differ from the typical images of the mesostructured samples (Figure 4a); stacking of 2D silica particles is observed, but, as previously commented, no information about the interlayer distances can be inferred. However, the AFM study shows a significant decrease in the averaged height values (ca. 1.4 nm) (Figure S7) when compared with the initial samples. This value is in good accordance with the *d* spacing observed in the XRD patterns of the collapsed surfactant-free samples, and confirms the loss of the paraffinic-type organization. These results (stability of the colloidal suspensions resulting from the dispersion of the layered matrices) suggest that, by optimizing the handling protocol, the UVM-Ln materials can be suitable precursors of nano/micro fillers in siliceous-polymer composites.

## 4. Conclusions

The main goal of the present work was to design a simple and reproducible method for the direct preparation of high purity silica-based micro/nanocharges. The “modified atrane route” allows us to obtain a wide family of layered mesostructured silica derivatives according to XRD, SEM–TEM, and AFM results. Moreover, the spectroscopic measurements combined with TGA–DTA and elemental analysis support the chemical homogeneity and good dispersion of the inorganic/organic functional groups, and also suggest that S^+^I^−^ and S^0^I^0^ mechanisms cooperatively work in the surfactant-assisted mesostructure formation. The main advantage of the resulting neutral-amine templated matrices (with regard to other natural or synthetic lamellar solids) lies in the ease of their manufacture; modification; expansion; and, very likely, adaptation to polymers, a set of processes that can be carried out in single one-pot procedures.

## Figures and Tables

**Table 1:** Selected physical parameters for UVM-Ln pure silicas.

Sample	n	d (001) ^a^/nm	Water ^b^/%	S^0^ Amine ^b^/%	S^+^ Amine ^b^/%	S^0^/S^+^ Ratio
UVM-Ln	8	2.74	3.5	26	13	2.0
UVM-Ln	10	3.35	3.4	28	19	1.5
UVM-Ln	12	4.16	4.9	33	21	1.6
UVM-Ln	14	4.92	2.2	33	24	1.4
UVM-Ln	16	5.48	1.4	41	24	1.7
UVM-Ln	18	5.91	1.7	44	26	1.7

^a^ Interlayer distance from X-ray powder diffraction (XRD) data. ^b^ Values determined from TGA.

**Table 2:** Selected physical parameters for UVM-Ln functionalized materials.

Sample	n	d (001) ^a^/nm	Si/Al ^b^/Nominal	Si/Al ^c^/Real	Si(Q/Si(T) ^b^/Nominal	Si(Q)/Si(T) ^d^/Real
Al-UVM-Ln	16	5.36	19	22	-	-
Al-UVM-Ln	16	5.33	9	7	-	-
Al-UVM-Ln	16	5.29	4	5	-	-
Al-UVM-Ln	16	4.03	2	3	-	-
Epoxy-UVM-Ln	12	4.13	-	-	12.5	4.5

^a^ Interlayer distance from XRD data. ^b^ Values from the reagent proportion in the starting solution. ^c^ Values determined by electron probe microanalysis (EPMA). ^d^ Values determined from ^29^Si MAS NMR.

## Supplementary Materials

The following are available online at http://www.mdpi.com/2079-4991/8/10/817/s1, Figure S1: XRD patterns for UVM-Ln solids, Figure S2: TGA curves for samples UVM-Ln, Figure S3: DTA curves for samples UVM-Ln, Figure S4: representative SEM image of UVM-Ln solids, Figure S5: Low-angle XRD pattern of the organically modified Epoxy-UVM-L12 sample, Figure S6: TEM image of the delaminated UVM-L16 sample, Figure S7: AFM images of the delaminated UVM-L16 sample.
