# Supplementary material for: Layered-Expanded Mesostructured Silicas: Generalized Synthesis and Functionalization
Source: Nanomaterials (Basel). 2018 Oct 11;8(10):817. doi: 10.3390/nano8100817 (PMC6215263; doi:10.3390/nano8100817)
Supplement: Supplementary file 1 [file nanomaterials-08-00817-s001.pdf]

Supplementary material

Layered-expanded Mesostructured Silicas: Generalized Synthesis and Functionalization

Pedro Burguete,[1] José Manuel Morales,[1] Lorenzo Fernández,[1] Jamal El Haskouri,[1] Julio Latorre,[1] Carmen Guillem,[1] Francisco Pérez-Pla,[1] Ana Cros,[1] Daniel Beltrán,[1] Aurelio Beltrán[1] and Pedro Amorós[1]*

[1] Institut de Ciència dels Materials, Universitat de València (ICMUV), P.O. Box 22085, 46071-Valencia (Spain)

* Correspondence: pedro.amoros@uv.es; Tel.: +34-963-543-617

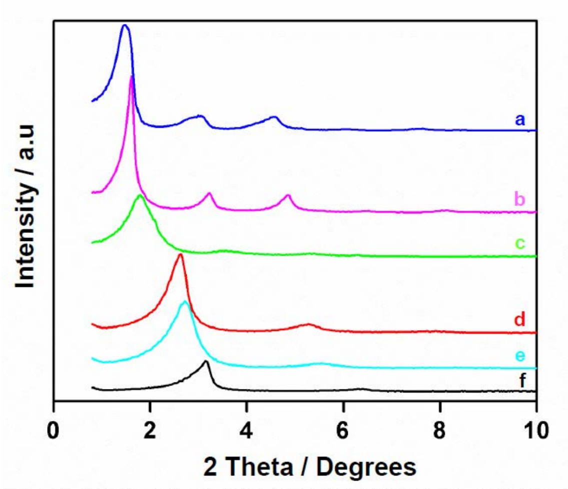

Figure S1. XRD patterns for UVM-Ln solids. (a) n=18, (b) n=16, (c) n=14, (d) n=12, (e) n=10 and (f) n=8.

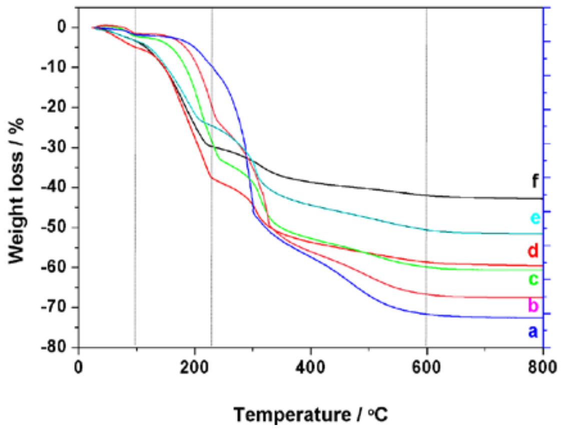

Figure S2. TGA curves for samples (a) UVM-L18, (b) UVM-L16, (c) UVM-L14, (d) UVM-L12, (e) UVM-L10 and (f) UVM-L8.

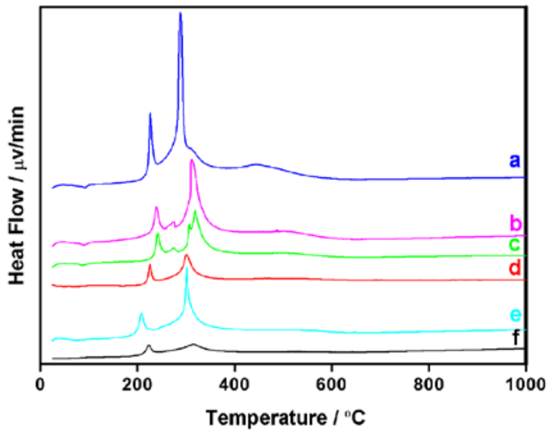

Figure S3. DTA curves for samples (a) UVM-L18, (b) UVM-L16, (c) UVM-L14, (d) UVM-L12, (e) UVM-L10 and (f) UVM-L8.

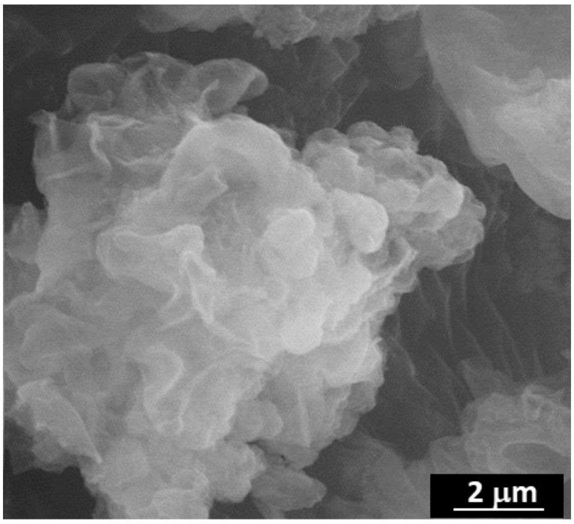

Figure S4. SEM image of UVM-L18 sample.

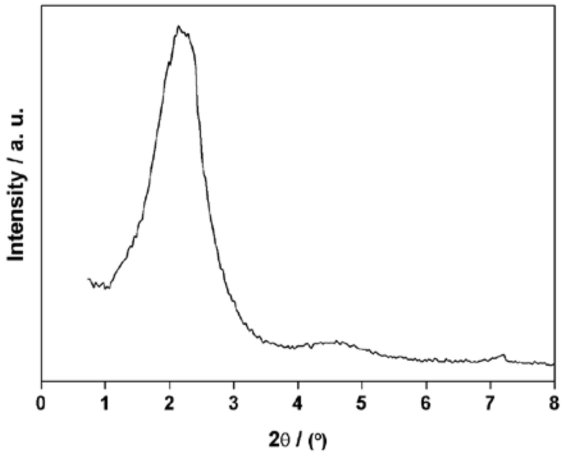

Figure S5. Low-angle XRD pattern of the organically modified Epoxy-UVM-L12 sample.

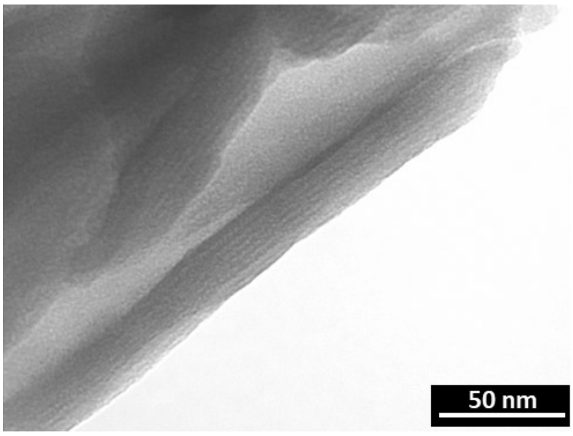

Figure S6. TEM image of the UVM-L16 sample dispersed in ethanol.

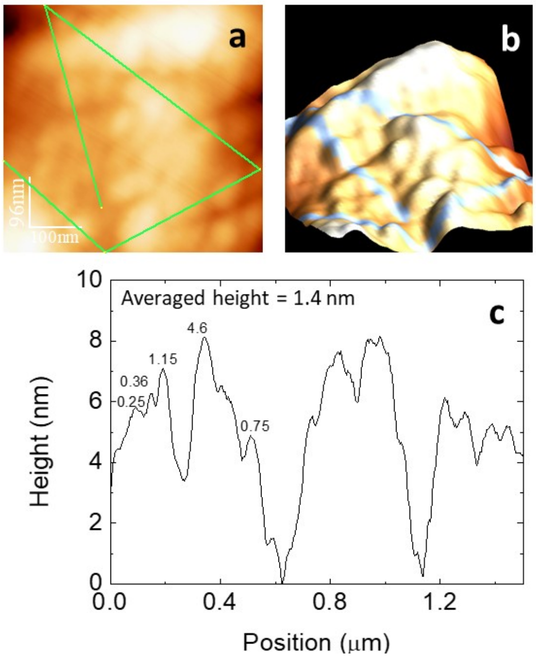

Figure S7. 2D (a) and 3D (b) AFM images of sample UVM-L16 after dispersion in ethanol. (c) Height vs position along the line indicated in the (a) image.
